# B Cell Response and Mechanisms of Antibody Protection to West Nile Virus

**DOI:** 10.3390/v6031015

**Published:** 2014-03-03

**Authors:** S. Kyle Austin, Kimberly A. Dowd

**Affiliations:** 1 Department of Medicine, Washington University School of Medicine, St. Louis, MO 63110, USA; 2 National Institute of Allergy and Infectious Diseases, National Institutes of Health, Bethesda, MD 20892, USA; E-Mail: dowdka@mail.nih.gov

**Keywords:** West Nile virus, flavivirus, humoral immunity, neutralizing antibody, epitopes, therapeutics

## Abstract

West Nile virus (WNV) has become the principal cause of viral encephalitis in North America since its introduction in New York in 1999. This emerging virus is transmitted to humans via the bite of an infected mosquito. While there have been several candidates in clinical trials, there are no approved vaccines or WNV-specific therapies for the treatment of WNV disease in humans. From studies with small animal models and convalescent human patients, a great deal has been learned concerning the immune response to infection with WNV. Here, we provide an overview of a subset of that information regarding the humoral and antibody response generated during WNV infection.

## 1. Introduction

West Nile virus (WNV) is a neurotropic flavivirus that has seen an emergence into new geographical regions in the last decade. Originally isolated from a patient in Uganda in 1937 [1], WNV was introduced into New York in 1999 and has since spread to the Pacific coast and through the Americas to Argentina. Since its introduction, WNV has become the leading cause of mosquito-borne encephalitis in the USA [2]. WNV now poses a risk to human health in North America, Europe, Africa, and the Middle East. 

A member of the *Flaviviradae* family, WNV is classified within the Japanese Encephalitis virus serocomplex. WNV exists in an enzootic cycle between mosquitos and birds, but humans and horses can become infected when bitten by an infected mosquito. While ~80% of infections are asymptomatic, WNV infection can cause a range of symptoms from a mild febrile disease to flaccid paralysis to lethal encephalitis. While the most severe symptoms generally manifest in the elderly and immunocompromised, healthy individuals can also experience severe disease. 

## 2. Virology and Pathogenesis

WNV has a positive, single-stranded ~11-kilobase RNA genome. The genome is encapsidated within multiple copies of the capsid (C) structural protein and enveloped in a lipid bilayer decorated by the two other structural proteins, membrane (M) and envelope (E). The infectious mature WNV particle is approximately 50 nm in diameter and has 180 copies of the E glycoprotein arranged in a *T = 3* quasi-icosahedral symmetry [3]. The viral lifecycle begins with attachment of the virus to a yet-to-be-identified cellular receptor. Several cellular proteins have been shown to interact with surface E proteins, including integrin α_v_β_3_ [4,5], DC-SIGN/ DC-SIGNR [6,7], and others [8], but none of these candidates were shown to be both necessary and sufficient for infection. The virus enters via clathrin-mediated endocytosis and traverses the lysosomal pathway [9]. As the endocytic vesicle containing the virus acidifies, structural rearrangement of E proteins occurs, allowing for the formation of E homotrimers and insertion of the fusion loop into the vesicular membrane [10,11,12]. The nucleocapsid is released into the cytoplasm of the cell, completing the first stage of infection. The WNV genome is translated as a polyprotein and subsequently cleaved by both viral and host proteases. The polyprotein encodes the three structural proteins (C; pre-membrane (prM); and E) and seven non-structural proteins (NS1, NS2A, NS2B, NS3, NS4A, NS4B, and NS5), the latter of which are involved in the replication complex. 

Animal models have aided our understanding of WNV pathogenesis in the absence of data for human pathogenesis. From these studies, WNV pathogenesis has been classified into three stages: initial infection and spread, peripheral viral spread, and neuroinvasion. Upon transmission of WNV from the bite of an infected mosquito, the virus is believed to infect and replicate within keratinocytes and skin-resident dendritic cells. It is thought that DC migration to the draining lymph node leads to the next phase of infection as the virus replicates and is disseminated into peripheral organs. It is currently unclear what the major cellular reservoir of viral infection and replication WNV uses, but subsets of DCs, macrophages, and neutrophils have been suggested. The final stage of WNV pathogenesis involves neuroinvasion and infection of the brain and spinal cord. The mechanism(s) by which WNV gains entry into the CNS is incompletely understood, but it is the translocation of the virus into the CNS that leads to lethal disease. 

## 3. WNV Structural Biology

**Figure 1:**
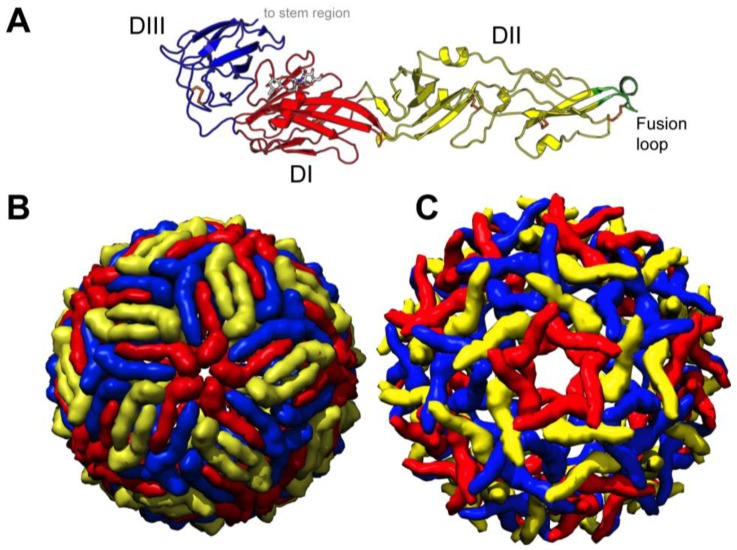
West Nile virus (WNV) structure. (**A**) Ribbon diagram of the crystal structure of WNV E ectodomain residues 1–400 (PDB 2HG0) [20] colored by domain: DI (red), DII (yellow), and DIII (blue). The fusion loop (residues 98–110) is shown in green, the six disulfides in orange, and the N-linked glycan at position Asn 154 is colored by atomicity; (**B**) Pseudoatomic cryo EM reconstruction model of the mature WNV virion (PDB 3J0B) [22]; (**C**) Pseudoatomic cryo EM reconstruction model of the immature WNV virion (PDB 2OF6) [24]. Each E monomer in the cryo EM models is colored according to its icosahedral symmetry location: 2-fold (yellow), 3-fold (blue), and 5-fold (red). Ribbon diagram rendered in PyMOL [25]. All cryo EM models were rendered in the program Chimera [26].

The E glycoprotein is the major flavivirus structural protein present on the viral surface, as well as the dominant target of neutralizing antibodies. The E glycoprotein is responsible for binding the host cellular receptor as well as endosomal fusion. The crystallographic structure of the E protein ectodomain of multiple flaviviruses has been determined [13,14,15,16,17,18,19,20,21]. Despite sharing only ~37% sequence identity, flavivirus E ectodomains share a generic structure of three subdomains stabilized by six conserved disulfide bonds (Figure 1A). The centrally located domain I (DI) is an eight-stranded β-barrel. Flanking DI is domain II (DII) that consists of two elongated loops, containing the conserved fusion loop (residues 98–110). Domain III (DIII) is an immunoglobulin-like fold linked to DI on the opposing side from DII. Glycosylation of the E glycoprotein is variable among flaviviruses. WNV E has a single N-linked glycosylation site at position 154, while dengue virus (DENV) and Tick-borne encephalitis virus have an additional N-linked glycan in E DII. The two α-helices following DIII are designated as the stem region, which is followed by two more α-helices in the transmembrane region. While there are no crystallographic models of these helices, we have been informed of their locations and functions from atomic modeling of cryo EM structures of flaviviruses [22,23]. Both sets of helices are found in anti-parallel arrangements; those of the stem region are amphipathic, interacting with both the phospholipid heads of the lipid bilayer and the viral structural proteins, while the hydrophobic transmembrane helices are inserted into the outer leaflet of the bilayer. 

The mature WNV virion is ~50 nm in diameter with 180 copies of the E glycoprotein smoothly arranged in a head-to-tail homodimeric fashion [3]. The homodimeric arrangement of the E protein shields exposure of the DII fusion loop at neutral pH. The symmetry of the E proteins creates three unique chemical environments according to the orientation into 2-, 3-, or 5-fold symmetry axes (Figure 1B). Immature WNV virions have a distinct structural composition relative to the mature virion. Unlike the mature virion, the immature virus maintains 180 copies of uncleaved prM protein, non-covalently associated with each E protein. These prM/E heterodimers form 60 trimeric spikes (a trimer of heterodimers) arranged with icosahedral symmetry ([27] and Figure 1C), with the DII fusion loop radiating outward from the virus center. prM is thought to cap the E fusion loop and prevent premature viral fusion as the newly assembled particle transverses the slightly acidic environment of the trans-Golgi network (TGN). The low pH environment of the TGN induces structural rearrangements of E proteins that expose a cleavage site within prM. After the prM protein is cleaved in the TGN by a host furin-like protease, the cleaved pr peptide remains associated with E by electrostatic forces, continuing to shield the viral fusion loop from premature fusion, until the virus is released into the extracellular environment [28]. In the neutral pH of the extracellular environment, the pr peptide dissociates from the mature particle, and the E protein assumes the homodimeric *T = 3* symmetry discussed above. Although cleavage of prM is required for the production of infectious particles [29], this cleavage event is not 100% efficient, resulting in the release of a heterogeneous mixture of mature, immature, and partially mature virions that retain varying levels of uncleaved prM [30,31,32]. At least a subset of partially mature WNV is infectious, indicating that an unknown threshold of prM cleavage is required for the transition from non-infectious (fully immature) to infectious virus [7]. The variability of this population has limited the use of traditional structural methodologies. Application of single particle reconstruction provided by cryo-electron tomography or an alternative method may yield some insight into the architecture of such particles. 

As the endosomal compartment matures during WNV entry, an acid-induced dissociation of the E homodimers occurs, facilitating the irreversible formation of a fusogenic E trimer. While the exact structural arrangements on the surface of the virion are unknown, crystallographic structures inform us that the formation of the trimer requires a considerable movement at the DI-III linker as DIII rotates ~36 degrees relative to DI/II [16,18,19]. These fusogenic spikes are oriented radially from the virus, with DIII and DI forming the base and the DII fusion loop clustering towards the host membrane. It has been proposed that an interaction between the stem region and transmembrane region stabilizes the trimer for eventual fusion of viral and cellular lipid and release of the nucleocapsid into the cytosol [33].

### Structural Flexibility of WNV

The structural flexibility of the E protein during maturation and fusion has been well established. However, studies over the last few years have revealed that the conformation of the virus, relative to antibody epitope exposure, is influenced by increasing the incubation time and temperature (discussed in Section 5.2.2). Indeed, recent cryo EM reconstructions of DENV after incubation at higher temperatures resulted in the transition to a “bumpy” virus, on which the E proteins are present in an expanded form as compared to the smooth virion stucture [34,35]. While incubation of WNV did not show these same structural transitions, the authors proposed that the higher body temperature of bird reservoirs at 43 °C may provide optimal conditions for a similar transition compared to that of the human body temperature. While epitopes that have limited exposure show a more drastic response to neutralization when both time and temperature is increased, readily available epitopes also show some increase in neutralization efficacy under the same conditions for both DENV [36,37] and WNV [38]. The full scope of the conformational ensemble for WNV is unknown. 

## 4. Humoral Immune Response to WNV

The humoral immune response plays a key role in the pathogenesis of West Nile virus infection. WNV-infected mice lacking B cells (μMT mice) show higher viral titers in the CNS and 100% mortality [39], presumably due to an inability to clear virus from the periphery. Administration of neutralizing monoclonal or polyclonal IgG immune serum to naïve WT mice provides complete protection from death while a subset of μMT mice experience delayed mortality. Furthermore, WNV infection of sIgM^−/−^ mice, which lack secreted IgM, but express cell surface IgM and can secrete other antibody isotypes, resulted in complete mortality [40]. Passive transfer of anti-WNV IgM from a mouse day four post-infection or anti-WNV IgG was able to blunt the dissemination of the virus in wild-type mice and mice lacking secreted IgM. These results demonstrated that the early anti-WNV IgM response (a) limits virus spread in the periphery and CNS and (b) the IgM titer on day four can predict survival outcome. However, passive antibody alone, while able to delay complete mortality, was not sufficient to protect RAG1^−/−^ mice, which lack both B and T cells, suggesting a role in viral clearance for T cell mediated immunity in WNV infection [41]. Beyond survival studies, the events that result in innate immune activation of B cells are incompletely understood. Sustained signaling through the type I interferon α/β-receptor has been shown to be required for initial activation of B cells in the lymph node, but not the spleen [42]. This study also showed that the activation of CD19+ B cells in the draining lymph nodes was polyclonal in nature, as the response was BCR-independent. A recent study involving a WNV vaccine model showed that mice lacking MyD88 had deficiencies in B cell activation, germinal center activation, and the generation of a B cell memory response [43]. 

While anti-WNV IgG clearly protects mice from rechallenge, the role of IgG in primary WNV infection is unknown. Based on the known kinetics of production of anti-WNV IgG isotypes (appearing between days 6–8), the virus enters the CNS (day 3) and is mostly cleared in the periphery [39]. In evaluating the possibility of antibody therapeutics, administration of immune human gamma globulin in the μMT mouse model of infection resulted in an increase in survival time, when given before day two post-infection [41]. WNV infection did show an increase of plasma cells in the brain of infected mice [44], perhaps suggesting a role for IgG in the CNS. Finally, multiple genetic deficiencies that affect anti-viral antibody priming, production, or trafficking (C3, C4, CD40, absence of CD4+ T cells, level of MHC class II expression, CD22) result in a concomitant decrease in antibody titers and survival during WNV infection [45,46,47,48,49].

### 4.1. Humoral Memory Response

Flavivirus infection has been shown to induce life-long humoral protection from future infection with the homologous virus [50]. The anti-WNV IgG response is essential in providing this protection during WNV infection [39,51,52,53] and vaccination [54,55,56]. However, limited data exists examining the memory recall response in WNV infection. Memory B cells (MBC) and long-lived plasma cells (LLPC) persist upon resolution of infection, and have been assigned non-redundant mechanisms of protection upon WNV challenge in immune mice [57]. Upon resolution of WNV infection, LLPCs, found primarily in the bone marrow, continue to secrete high-affinity antibody specific for a single immunodominant epitope found in the initial viral E protein. However, the majority of antibodies from MBCs were able to recognize both the immunizing immunodominant epitope, as well as a mutant epitope these mice had not been exposed to. These results suggest that the antibodies produced by MBCs serve to neutralize a potential future challenge by variant WNV that might escape neutralization by the high affinity LLPC antibody. Indeed, a recent study shows that mice given a vaccine for the related Japanese Encephalitis virus are protected from WNV lethality in a MBC adoptive transfer model [58], demonstrating a cross-protective role for MBCs in flavivirus infection. It was demonstrated in a WNV vaccine model in mice lacking TLR3 that while the development of MBCs was not affected, the maintenance of germinal centers and LLPCs were negatively impacted [43].

### 4.2. Epitopes Targeted by WNV-Specific Antibodies

**Figure 2:**
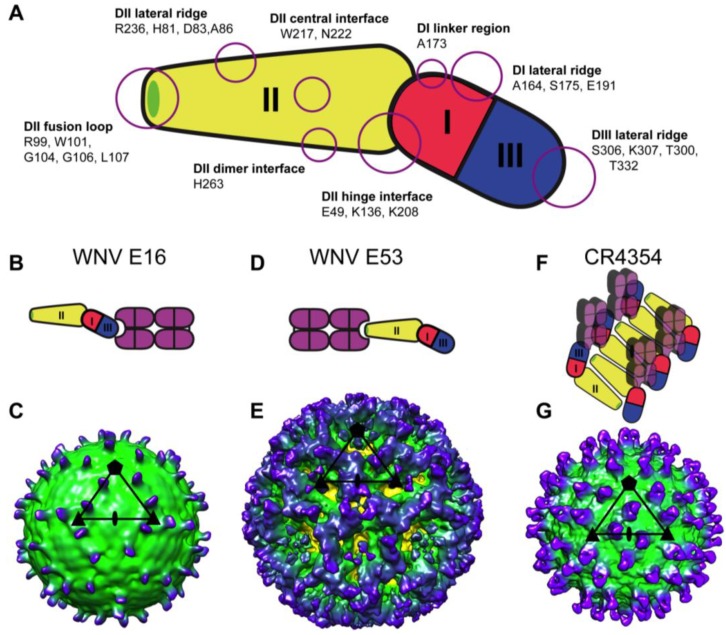
Antigenic structure of WNV. (**A**) Cartoon diagram of WNV E, colored by domain, labeling the location of neutralizing epitopes within E and associated residues; (**B**) Cartoon diagram of WNV E16 Fab engagement of DIII; (**C**) Cryo EM reconstruction of E16 Fabs bound to mature WNV virion (EMD_1234) [69]; (**D**) Cartoon diagram of WNV E53 Fab engagement of DII and fusion loop; (**E**) Cryo EM reconstruction of E53 Fabs bound to immature WNV virion (EMD_5103) [61]; (**F**) Cartoon diagram of CR4354 Fab engagement of a complex epitope between adjacent E monomers; (**G**) Cryo EM reconstruction of CR4354 Fabs bound to mature WNV virion (EMD_5190) [68]. Cartoon Fabs shown in purple, and WNV E colored as in Panel A. Cryo EM images colored according to distance from center of virus particle from lighter colors to darker. Black shapes on cryo EM figures identify axis symmetry: pentagon, 5-fold; triangle, 3-fold; ellipse, 2-fold.

The E protein, which comprises the majority of the virion surface, represents the major target of neutralizing antibodies to WNV infection. Antibody epitopes have been identified on all three domains (DI-DIII) [24,52,53,59,60,61,62,63,64], with the most potent neutralizing antibodies focused on a discontinuous epitope on the lateral ridge of DIII (DIII-LR) [52,64,65]. E16, an extensively studied DIII-LR mAb, is capable of neutralizing WNV infection at picomolar concentrations [66]. Locations within the E protein and specific residues involved in binding by various WNV neutralizing antibodies are shown in Figure 2. Epitope mapping of WNV-specific mAbs has been performed by numerous methods including structural analysis, identification of neutralization escape mutants, and binding assays utilizing various forms of the E protein (*i.e.*, linear peptides, soluble or yeast displayed forms of the E ectodomain or truncated E subdomains, intact virions or sub-viral particles) [67]. While individual epitopes have generally mapped to residues located spatially proximal to each other within specific E domains, evidence of more complex epitopes has been observed. For example, a subset of human WNV immune sera found to react with recombinant, full length E, but not polypeptides representing linear, 30 amino acid segments of the E protein, highlights the presence of antibodies that bind complex epitopes within a single E protein [51]. In a separate study, two mAbs isolated from B-cells of WNV-infected humans, CR4348 and CR4354, were found to bind intact sub-viral particles and virions but not to recombinant E or DIII alone, suggesting these antibodies bind epitopes that include residues from neighboring E proteins [53,62]. Indeed, the epitope for CR4348 maps to two amino acids (T208, H246) that are distal from one another on a single E protein, but are located within close proximity along the DII dimer interface of two antiparallel E proteins. Similarly, structural studies confirmed that CR4354 binds a discontinuous epitope that includes amino acids from neighboring E proteins from distinct dimers, and that this antibody acts by crosslinking the six E proteins located within a raft (Figure 2F,G) [68]. Both of these antibodies neutralize at a post-attachment step, indicating a mechanism by which the binding across multiple E proteins inhibits pH-mediated structural changes required for viral fusion [62].

Antibodies against WNV proteins other than E have been identified. As introduced above, a subset of infectious WNV virions retain varying levels of uncleaved prM. Antibodies that bind to prM have been identified in WNV immune sera [51,70], and prM antibodies have been isolated from both mice and humans [71,72]. As reported for other flaviviruses, WNV antibodies specific for prM generally display weak neutralizing activity and limited protection *in vivo* [71]. This likely stems from the limited number of prM molecules present on the surface of partially mature virions, the effects of which will be discussed in detail below. Antibodies specific for the non-structural protein NS1 that demonstrate protective activity from WNV infection *in vivo* have been described [73]. Prophylactic treatment with some NS1 mAbs protected mice against lethal WNV infection, despite the fact that NS1 is not associated with the virion itself. NS1 antibodies are hypothesized to bind cell-surface expressed NS1 on infected cells and result in phagocytosis of infected cells through interactions with Fc-γ receptors (FcγR) expressed on macrophages [74]. Finally, antibody responses directed at NS3 and NS5 [70,75], as well as capsid [51] have been observed, but little is known regarding their importance in protecting against WNV infection. 

## 5. Mechanisms of WNV Neutralization

Studies with WNV indicate that neutralization is governed by a stoichiometric threshold [31,66,76,77]. This requirement is consistent with a “multiple-hit” model of neutralization and implies that binding by a threshold number of antibody molecules is sufficient to disrupt critical steps during the infection process such as attachment or fusion with a target cell [78,79]. Based on studies with E DIII-LR specific mAbs, including E16, the stoichiometric threshold for WNV neutralization was estimated to require binding by ~30 antibody molecules per virion [66]. It remains to be determined whether a similar threshold of 30 antibodies applies to neutralization involving epitopes located elsewhere on the E protein. E16 neutralizes infection primarily by blocking viral fusion [64,80,81]; different stoichiometric thresholds may govern neutralization by antibodies that block infection by distinct mechanisms, such as blocking attachment. The stoichiometric threshold may also differ for antibodies that are capable of bivalent binding, which has been reported for a recently characterized DENV DIII-specific mAb [82]. Regardless, studies with diverse WNV-specific antibodies indicate that all act within the framework of a multiple-hit model of neutralization. 

### 5.1. Antibody Affinity and Epitope Accessibility Govern WNV Neutralization

Two critical factors determine whether the required threshold for antibody binding is met. Antibody affinity controls the fraction of epitopes occupied by antibody at a given concentration [83]. Differences in neutralization by two antibodies that bind a similar epitope can often be explained by differences in binding affinity. Similarly, mutating the virus in such a way that reduces the binding affinity of a mAb has the same effect. For example, mutation at E residue T330I results in >80% reduction in binding by E16 [52], which translates into a requirement for higher concentrations of antibody to neutralize infection [76,84]. Cryo EM and crystallography models indicate that E16 binds to 120 of 180 total E proteins on the mature virion; binding is precluded from the 60 epitopes located proximal to the 5-fold symmetry axes due to steric constraints with neighboring E proteins (Figure 2B,C) [64,69]. In the context of antibody affinity, neutralization of WNV therefore occurs when E16 is docked on the virus at a relatively low occupancy (30 of 120 possible epitopes); 25% of available epitopes must be bound by E16 for neutralization to occur. However, occupancy requirements for neutralization can significantly increase for epitopes that are exposed fewer times on the surface of the virion. For example, some DIII-specific antibodies that bind outside of the LR must occupy essentially 100% of available epitopes to reach the neutralization threshold [66]. In fact, many weakly neutralizing WNV-specific antibodies may behave so not because they bind with low affinity, but because their cognate epitope is not readily available for binding. Limited epitope accessibility likely explains why the majority of antibodies that bind epitopes within the DII fusion loop (DII-FL) are characterized by weak neutralizing potency [24,53]. The DII-FL specific mAb E53 binds WNV with similar affinity as E16, yet is significantly less neutralizing [31]. The crystal structure of the E53 Fab fragment bound to immature WNV illustrated a preferential ability of E53 to bind to E trimers found in the immature virus; the DII-FL epitope is not solvent accessible in the context of the E dimers present on mature WNV (Figure 2D,E) [61]. 

### 5.2. Factors That Modulate WNV Epitope Accessibility

Epitope accessibility has emerged as a critical factor that governs WNV neutralization. Mechanisms by which the virus can increase the number of epitopes docked by antibody increase the chances that neutralization requirements are met. Characteristics of WNV that modulate epitope accessibility, and their effects on neutralization potency will be discussed in detail below, using the mAbs E16 and E53 as examples. In the context of this review, an “inaccessible” epitope is one that cannot be engaged by antibody; this applies to cryptic epitopes that are not displayed on the surface of the virion, as well as those that are solvent accessible but precluded from binding by steric constraints.

#### 5.2.1. Structural Heterogeneity of WNV due to Inefficient Maturation

**Figure 3:**
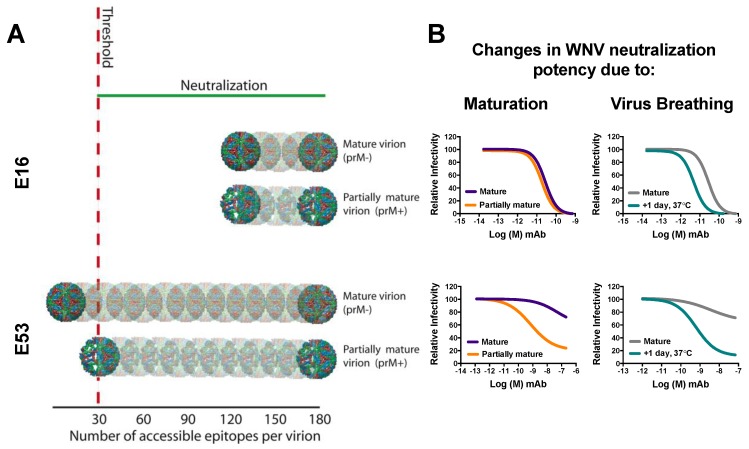
Epitope accessibility affects WNV neutralization. (**A**) Model of how the extent of maturation and virus breathing affect neutralization potency of the mAbs E16 and E53 against WNV; (**B**) Neutralization dose-response curves supporting the model presented in panel (a). Left panels show neutralization curves of E16 or E53 against WNV produced under conditions that either promote or reduce the extent of maturation (“Mature” WNV generated in the presence of an overexpression of furin to promote prM cleavage (purple curves), *versus* “Partially mature” WNV generated in the presence of the weak base NH_4_Cl to inhibit the structural rearrangements required for prM cleavage (orange curves), respectively). Right panels show neutralization curves of mature WNV incubated either for 1 h at room temperature (grey curves), or an additional ~24 h at 37 °C (teal curves) before infection of cells.

Mapping studies identified a subset of WNV-specific antibodies with the potential to neutralize infection that bound epitopes not predicted to be accessible on the mature form of the virus [24]. One explanation for this inconsistency is the structural heterogeneity of WNV virions released from infected cells with respect to maturation, as introduced in Section 3. Partially mature WNV provide a heterogeneous landscape for antibody binding, as these viruses display E proteins that resemble the homodimers associated with the mature form of the virus and the prM-associated trimers found on the immature form. Because of differences in epitope accessibility between these E protein arrangements, the neutralizing potency of certain classes of antibodies is modulated by the extent of maturation [31,32]. While the extent of maturation can be artificially modified *in vitro* [7,31,38,85], there is evidence of natural variation in prM cleavage when using different cell types to generate WNV [32]. Using populations of infectious WNV representing the far ends of the maturation spectrum, E53 is relatively incapable of neutralizing mature WNV due to the cryptic nature of the DII-FL epitope, but becomes more potent as the levels of uncleaved prM retained on the virus increase (Figure 3). As described in Section 5.1, this occurs because E trimers associated with the immature form of the virus display the DII-FL epitope in a surface accessible position allowing E53 binding [61]. A hallmark of most maturation state-sensitive antibodies is the presence of a “resistant fraction” of infectious virus observed in neutralization assays even in the presence of saturating concentrations of antibody. In the case of E53, these particular viruses have lower levels of uncleaved prM (are more mature), and thus the DII-FL epitope is displayed an insufficient number of times to meet the required threshold for neutralization [31]. Interestingly, the pattern of epitope accessibility that governs all maturation sensitive WNV antibodies identified to date is such that maturation is only associated with decreases in epitope exposure. Antibodies specific for epitopes that become more accessible as maturation proceeds to completion have not been identified; the basis for this is unknown. Not all antibodies are sensitive to the maturation state of the virus. The E16 DIII-LR epitope is equally solvent accessible on both the mature and immature forms of the virus, resulting in identical neutralization potency regardless of the maturation status of the virus population (Figure 3) [31,85]. Unexpectedly, the neutralization potency of the DIII-LR specific mAb E33, which has a similar binding footprint as E16, was found to be maturation-state sensitive. It was discovered that steric constraints arising from the positioning of the Fc-portion of antibody molecules bound to the mature, but not the immature form of the virus were responsible for the maturation state dependence of E33. These steric constraints resulted in a preferential decrease in neutralization potency of E33 against mature WNV, and could be alleviated by using Fab fragments [86]. Thus, at least two mechanisms exist by which the extent of maturation modulates WNV antibody neutralization. 

#### 5.2.2. Virus Breathing Increases Epitope Accessibility

As introduced in Section 3.1, flavivirus virions explore an ensemble of conformations at equilibrium, with the potential to increase epitope exposure. Current cryo EM structures of virions likely represent only the average or preferred conformation sampled in this process, referred to as structural dynamics or virus “breathing”. Studies with WNV and a panel of mAbs specific for all three E domains indicated that structural dynamics affects neutralization of all antibodies; kinetic increases in neutralization were observed when virus and antibody were incubated for increased lengths of time, or at increased temperature [38]. That the rate of structural dynamics increases at elevated temperatures suggests a mechanism by which the induction of a fever in response to infection can result in increased neutralization potency, and has implications regarding the transmission of WNV from insect to human hosts that maintain distinct body temperatures. The effect of structural dynamics on neutralization of mature WNV by E16 and E53 are shown in Figure 3. Initially E53 is incapable of neutralizing mature WNV, however large increases in potency are observed with increased incubation time. Since the DII-FL epitope is buried on the surface of the cryo EM structure of the mature virion, these increases provide direct evidence of dynamics-mediated epitope exposure. E16, although already capable of potently neutralizing WNV in a standard assay, still displays modest dynamics-mediated increases in neutralization. As discussed in Section 5.1, although the DIII-LR epitope is surface accessible, in the context of the mature virion, E16 is predicted to bind only 120 of 180 total sites due to steric constraints. By sampling structural conformations that alleviate steric constraints and allow for at least some of these remaining epitopes to be docked by antibody, dynamics-mediated increases in neutralization can occur for even the most potent WNV antibodies.

Ongoing studies indicate a correlation between flavivirus structural dynamics and the intrinsic stability of virus in solution. Some pathways of virus breathing may result in a virus that is “stuck” in a non-infectious state, which manifests as a loss of infectivity. Interestingly, the extent of WNV maturation impacts the intrinsic decay rate of the virus; fully mature virus decays (loses infectivity) at a slower rate than populations of WNV that retain high levels of prM [87]. WNV virions that display distinct conformations of E proteins likely have separate “breathing” pathways, adding yet more complexity to our understanding of how epitope accessibility affects WNV neutralization. 

## 6. Antibody Fc-Region Effector Functions

While engagement of the virion by a sufficient number of WNV-specific antibody molecules can directly neutralize infection by blocking binding or fusion, the non-antigen binding Fc-portion of an antibody is capable of modulating infection by interacting with additional proteins present *in vivo*. Through the classical pathway of complement activation, C1q molecules that consist of six globular heads form multivalent interactions with the Fc-portions of either single pentameric IgM or multiple IgG antibodies docked on the surface of a virus. C1q opsonization triggers a cascade of cleavage events that result in the formation of the membrane attack complex (MAC) capable of viral lysis [88]. However, while the presence of complement proteins in serum has been shown to increase neutralization potency against WNV infection *in vitro*, this does not seem to be dependent on MAC induced viral lysis. Neutralization assays performed in the presence of serum from mice deficient in various complement components demonstrated that C1q, but not C3 or C5 (which act downstream from C1q in the complement cascade), was necessary for the increase in neutralization [77]. Addition of exogenous C1q to neutralization assays performed *in vitro* results in increased neutralization potency. Mechanistically, binding of C1q to antibody-virus complexes effectively lowers the stoichiometric threshold required for neutralization, and involves the ability of C1q to crosslink antibodies docked on the virion surface. Mannose binding lectin (MBL), a component of the non-classical lectin pathway of complement activation, has also been implicated in WNV neutralization. Similar to the mechanism of C1q, this inhibition does not require downstream activation of the complement cascade. However, the activity of MBL in limiting WNV infection is antibody independent; MBL binding to the virion surface may directly neutralize infection by inhibiting fusion [89,90].

A recent study demonstrated that Fc effector functions may enhance the *in vivo* protective effects of antibodies that are poorly neutralizing *in vitro* [32]. The ability of the DII-FL mAb E28 to modestly protect mice from lethal WNV infection when administered one day prior to infection was found to be dependent on antibody Fc interactions with both FcγR and C1q, as a larger proportion of mice lacking either or both of these molecules succumbed to infection. Additionally, the protective effect of E28 in WT animals was diminished when an aglycosyl version of the antibody was substituted that could no longer engage FcγR or C1q. While the *in vivo* protective effects of DII-FL specific antibodies were greatly inferior to DIII-LR specific antibodies, which resulted in survival of even the FcγR/C1q deficient mice, this study highlights a mechanism by which poorly neutralizing antibodies may be protective *in vivo*. This is of particular importance based on the finding that the human antibody response is overwhelmingly directed at epitopes within the DII-FL, as discussed in Section 7 [53,60,63].

The Fc-region of antibody molecules has also been implicated in antibody-dependent enhancement of infection (ADE), a phenomenon that describes an increase in virus infection in the presence of sub-neutralizing antibody concentrations. In this process, antibody-virus complexes infect cells through uptake by Fc receptor interactions [91]. A presumed role for ADE in enhancing DENV infection and disease severity *in vivo*, primarily during secondary infections, is a constant concern in the development of a much-needed vaccine [92]. While ADE can be observed for WNV *in vitro*, clinical manifestations in human infections are not readily apparent [76,93,94]. Regardless, *in vitro* studies with WNV indicate that ADE and neutralization are related to one another by the number of antibodies bound to the virus. ADE is governed on the lower end by the number of antibody molecules required to mediate cell attachment, and on the upper end by the neutralization threshold. For DIII-LR antibodies, ADE is estimated to occur when between 15 and 30 antibody molecules are bound to the virus [66]. Since C1q effectively reduces the stoichiometric threshold required for neutralization, the presence of C1q can abolish the potential for ADE by some antibodies, and may in part explain the lack of enhancement observed *in vivo* [46]. Antibodies that bind epitopes exposed few times per virion would presumably be good candidates for initiating ADE. Studies have suggested that fully immature WNV and DENV can be rendered infectious by Fc-receptor mediated uptake of virus-antibody complexes [95,96,97]. However, whether furin cleavage acts to cleave prM on entering WNV virions is unclear [85]. Regardless, partially mature flaviviruses remain candidates for ADE by weakly neutralizing antibodies, such as those that bind prM.

## 7. Understanding the Human Polyclonal Response to WNV

Most data concerning the antibody response to WNV are from mouse studies, which indicated that a large portion of the humoral response was directed at epitopes within the DIII-LR [52,65,98,99,100]. However, there appears to be differences in the composition of the mouse antibody epitope repertoire relative to that of humans [53,60,63]. Using E protein variants, one study calculated that while the DIII-LR was the major target of neutralizing antibodies produced in mice, these antibodies were rare in serum from WNV-infected convalescent patients. Indeed, in this study, the major human target was the region containing the DII-FL [63]. Yet other studies have identified potently neutralizing antibodies from humans binding complex epitopes in the DI-DII linker region, such as those described in Section 4.2 [53,62]. Furthermore, studies with WNV and DENV human sera suggest that antibodies targeting the DIII-LR are functionally inconsequential [31,63,101,102]. Further studies using large panels of human samples are needed to both identify the immunodominant target(s) of the human antibody response, as well as the role of DIII-specific antibodies.

## 8. Progress of WNV Vaccine and Therapeutics

There are no approved human vaccines against WNV. Both subunit and attenuated vaccine candidates have entered into phase I and phase II clinical trials [103], yet none have progressed to phase III trials. Some data evaluating the targets of potently neutralizing human antibody responses in flaviviruses suggest these targets are complex epitopes formed by viral symmetry [62,68,102]. In light of this data, vaccines that include or will encode the genes necessary to form the full viral particle should be examined.

As there is currently not an approved vaccine for the prevention of WNV, one possible treatment is passive administration of neutralizing antibody to patients affected by WNV disease. In studies with mice and hamsters, the E16 antibody was capable of protecting the majority of these animals up to five days post-infection with WNV [52]. Indeed, several studies have shown that human immune serum and intravenous immunoglobulin (IGIV) can protect mice from lethal WNV challenge [104,105,106]. Protection after the onset of clinical symptoms and WNV encephalitis is likely a requisite of passive antibody immunotherapy for a possible treatment of infection. There are a growing number of examples of solid organ transplant related WNV infections [107,108,109,110,111,112]. In one example, a patient receiving a liver from a WNV-infected donor did not develop anti-WNV IgM until 26 days after the transplant, and failed to develop an IgG response >4 months after seroconversion [110]. While it is inconclusive that the IGIV protected this patient from fatal WNV infection, this population is generally receiving immunosuppressant drugs to prevent organ rejection, and at high-risk for severe WNV disease. Additional studies are needed beyond the non-controlled case studies described by the literature. Currently, immunotherapeutics have progressed to phase II clinical trials for the IVIG Omr-IgG-am consisting of high anti-WNV titers of convalescent Israeli blood donors and that of the monotherapy of MGAWN1, the humanized equivalent of the mouse E16 antibody [113,114]. 

## 9. Conclusions

The need for WNV therapeutics and vaccines remains a high priority, as WNV continues to pose a significant public health threat in various regions of the world. The critical role of the WNV-specific humoral response and antibodies in controlling infection underscores the need for a greater understanding of this arm of the immune response. Considerable progress has been made towards mapping WNV epitopes within the E protein, and has led to the identification of potent neutralizing antibodies that have been adapted into candidates for therapeutic use. However, many questions remain unanswered and new questions have arisen from recent discoveries, including: (i) what is the role, if any, of IgG in primary infection; (ii) what is the role of anti-prM antibodies in infection; (iii) how does the structural heterogeneity of WNV (due to maturation and structural dynamics) impact neutralization *in vivo*; (iv) why does the human response preferentially target non-DIII-LR epitopes and what are the immunodominant epitopes; and (v) what are the early activating signals for B cells. The ability to understand WNV neutralization in quantitative terms and apply this knowledge towards dissecting the polyclonal response to WNV will aid in the design of future vaccine candidates.
